# Association between induced abortion and suicidal ideation among unmarried female migrant workers in three metropolitan cities in China: a cross-sectional study

**DOI:** 10.1186/s12889-018-5527-1

**Published:** 2018-05-15

**Authors:** Mengyun Luo, Xueqin Jiang, Ying Wang, Zezhou Wang, Qiuming Shen, Rui Li, Yong Cai

**Affiliations:** 1 School of Public Health, School of Medicine, Shanghai Jiao Tong University, Shanghai, 200025 People’s Republic of China; 2 Department of Public Health Sciences, University of Rochester School of Medicine and Dentistry, Rochester, NY 14642 USA

**Keywords:** Induced abortion, Suicidal ideation, Female migrant workers, Mental health

## Abstract

**Background:**

Despite reports of mental health issues, suicidality has not been closely examined among the migrant population. The association between induced abortion and suicidal ideation is unknown among unmarried female migrant workers of reproductive age in China. This study aims to examine induced abortion and suicidality among the Chinese migrant population.

**Methods:**

We recruited 5115 unmarried female migrant workers during 2015 to 2016 from Shanghai, Beijing and Guangzhou, and collected demographic, psychosocial, reproductive and mental health information using structured questionnaires. We used logistic regression models to examine the association between lifetime induced abortion and suicidal ideation during the past year among the subjects.

**Results:**

Overall, 8.2% of the subjects had suicidal ideation during the past year, and 15.5% of the subjects experienced induced abortion. Induced abortion was associated with nearly twice the odds of having past-year suicidal ideation (Odds ratio, OR = 1.89; 95% confidence interval, CI: 1.46, 2.44) after adjusting for age, education, years in the working place, tobacco use, alcohol consumption, daily internet use, attitude towards premarital pregnancy, multiple induced abortion, self-esteem, loneliness, depression, and anxiety disorders. The association was stronger in those aged > 25 (OR = 3.37, 95% CI = 2.16, 5.28), with > 5 years of stay in the working place (OR = 2.98, 95% CI = 2.02, 4.39), the non-anxiety group (OR = 2.28, 95% CI = 1.74, 3.00), and the non-depression group (OR = 2.94, 95% CI = 2.08, 4.15).

**Conclusions:**

Induced abortion was associated with increased odds for suicidal ideation among the unmarried female migrant workers in urban cities in China. More attention should be paid to the mental health of the population.

## Background

As one of the hallmarks for global development, migration is and will continue to be associated with health issues. Internationally, migration is a process during which a person moves from one cultural setting to another [1], while internal migration usually occurs within a nation, and in China is characterized by the overwhelming rural-to-urban migration. It was estimated that there were 73 million migrants living in the World Health Organization (WHO) European Region in the early 2016 [2]. In China, the number tripled, corresponding to 247 million internal migrants living in the places different from their household registration, and the number will further increase to over 2 billion by 2020 [3]. Unlike most permanent residents who can enjoy the benefit of local public services and welfare systems, migrants, classified as temporary residents, generally experience disparities in their living conditions, including the exclusion from access to some medical services and social benefits [4], and experienced stigma and discrimination [5].

Between January and May 2010, twelve young migrants working in Foxconn Technology Group in Guangzhou attempted suicide among whom 10 died [6, 7], arousing public attention to the poor mental health of the migrant workers in China. It was reported that younger age (18–35 years) was a risk factor while male gender and being married were protective factors for depression and anxiety disorders in the general population [8–10], and therefore unmarried female migrant workers of reproductive age might be under higher risk for developing mental health problems including suicidal ideation, which is defined as any self-reported thoughts of engaging in suicide-related behavior [11]. Although not sharing exactly the same risk factors as suicide, suicidal ideation is an established risk factor for suicide [12] which is the second leading cause of death among 15–29 year olds globally [13], and different from Western countries where the suicide mortality is constantly higher in males, in China the gender difference in suicide rate is not salient despite a shift towards the Western trend over time, and more suicidal attempts occur among females especially in rural areas [14]. Several studies reported higher risk for suicidal thoughts and attempts among migrant workers [15, 16], while others suggested similar risks compared to urban dwellers [10]. In a study conducted among rural young Chinese, the prevalence of serious past-year suicidal ideation was 1.3% among the migrant group and 3.0% among the non-migrant group [17]. Another one reported 9.2% past-year suicidal ideation among women of reproductive age working in entertainment venues in the capital city of one eastern Chinese province [18]. However, there is still a dearth of data on unmarried female migrant workers in China.

Nowadays, females account for nearly half of the whole internal migrant group in China, and the number is steadily growing [19]. Most of them are unmarried women of reproductive age. Disparities between non-migrants and migrants in reproductive and maternal health, as well as family planning services accessibility result in higher incidences of induced abortion, caesarean section, and pregnancy complications [20]. As was reported, in China, nearly 15% of the unmarried female migrants had an unwanted pregnancy and 95% of them had an abortion [21, 22], among whom half had repeated abortions [23]. These self-reported data may be an underestimate since premarital sex is generally regarded as shameful in the sexually conservative Chinese culture. Fetal loss is a traumatic experience and studies have shown that abortion was correlated with increased risk for subsequent mental disorders [24–26], and suicidal behaviors [27, 28]. Although induced abortion-related suicidality among unmarried female migrant workers was rarely reported, the risk might increase as a result of physical impairment following abortion, and multiple psychosocial stresses which could be either innate in the population or related to induced abortion [29].

As there were few studies targeting the mental health of unmarried female migrant workers, our study aimed to understand the mental health of the population in big cities in China. We examined the association between lifetime induced abortion and suicidal ideation during the past year, and further explored whether the association, if any, varied between different subgroups. We hypothesize that (1) lifetime induced abortion was positively associated with past-year suicidal ideation; (2) the association between induced abortion and suicidal ideation differed by age group, hometown, education attainment, length of stay in the city, attitude towards premarital pregnancy, and the presence of depression and anxiety disorders.

## Methods

### Design and study population

The study was conducted in Shanghai, Beijing and Guangzhou, three of the largest metropolitan cities attracting a great number of migrants in China. Approximately 20 million migrants in China are currently residing in these three cities [30–32]. The eligible criteria for the study subjects were unmarried females aged over 18 years and working in the place different from their household registration as migrants. The exclusion criterion was the inability to read or answer questions (e.g., dementia, difficulties with the language).

Between June 2015 and March 2016, we recruited participants using the convenient sampling method. Assuming a prevalence of past-year suicidal ideation in rural-to-urban female migrant workers of 8% [18, 33, 34], using α of 0.05, and a relative error for sampling of 0.1, we calculated a required sample size of 5500 to allow for the non-response rate of 20%. In the first stage, we selected two factories from each city where female migrants congregated with the assistance of the officials from the local centers for disease control and prevention (CDC). In the second stage, we selected individuals compatible with the inclusion criteria from each factory with the help of the factory managers.

We conducted the survey with self-administrated questionnaires. We trained human resources specialists of the factories and senior undergraduate medical students from each city as investigators prior to the investigation. Each interview lasted approximately 30 min in a private room of each factory. We compensated each participant with 30 RMB ($4–5 USD) in cash after they completed the questionnaire. The Ethics Committee approved the survey. We obtained written informed consent from all the participants following a clarification of the objectives and the procedure of the study, as well as the potential risks and benefits of participation before enrolling the participants.

### Instruments and measures

#### Psychosocial variables

Psychosocial information included behavioral variables, attitudes toward premarital pregnancy and multiple abortion, as well as self-esteem and perceived loneliness. Behavioral variables included tobacco use, alcohol assumption and daily internet use within the past month which were assessed by four-point Likert scales questions asking the frequency of these behaviors (1 = never, 4 = everyday). We only considered subjects as non-smokers or non-drinkers if their answer was ‘never’ to the corresponding questions while we considered subjects as daily internet users if their answer was ‘every day’. We assessed the attitudes towards premarital pregnancy and multiple abortion with two questions: “Do you agree with premarital pregnancy” and “Do you agree with multiple induced abortion”, with the answers ranging from 1 (strongly agree) to 5 (strongly disagree). We considered subjects holding negative attitude towards premarital pregnancy or multiple induced abortion if their corresponding answers were “disagree or strongly disagree”, and other answers (“strongly agree, agree or doesn’t matter”) as having positive attitude. We measured self-esteem using the 10-item, Likert-type Rosenberg Self Esteem Scale [35, 36]. After reversing the negative items, we calculated the total score with a higher score indicating higher self-esteem (Cronbach’s alpha = 0.772; range 0–30). We further categorized the subjects into high- and low-level self-esteem groups with the cutoff point of 15 [37]. We measured the inner feeling of loneliness and the experience of human relationships with the 8-item ULS-8 Scale [38], a simplified version of the 20-item UCLA Loneliness Scale [39, 40]. Each item ranged from 1 (never) to 4 (most of the time), with a higher summed score indicating a higher level of loneliness (Cronbach’s alpha = 0.694; range 8–32). We defined the group with significant loneliness as those with a total score greater than 17, which was the 75th percentile in our study.

#### Mental disorder variables

We measured depressive symptoms using the 20-item Likert-type Center for Epidemiologic Studies Depression Scale (CESD) [41, 42]. A higher summed score indicated higher depression severity (Cronbach’s alpha = 0.865; range 0–60). According to the literature, we adopted 20 as the diagnosis value [43]. We measured anxiety with the 7-item Likert-scale Generalized Anxiety Disorder (GAD-7) [44, 45], with a higher summed score indicating more severe generalized anxiety (Cronbach’s alpha = 0.866; range 0–21). We defined those with the total score greater than 10 to be the anxiety group [44].

#### Reproductive health

For the reproductive health of the subjects, we asked three questions (Have you had premarital sex? Have you had unplanned pregnancy? Have you had the experience of induced abortion?). Subjects gave the response with “yes = 1” or “no = 0” for each question.

#### Suicidality

We measured suicidal ideation, suicidal plan and suicidal attempt during the past year using the questions “Have you ever.. . thought about killing yourself?”; “.. . made plans to kill yourself? ”; and “.. . failed to kill yourself?” within the past year with the response “yes = 1” and “no = 0” for each question. For this study, we focused on the association between induced abortion and suicidal ideation due to heterogeneity in the risk factors across the spectrum of suicidality.

### Statistical analysis

The quality of data was ensured by the double entry process. Cases with any missing data were excluded from the analysis. We used Chi-Square test to examine the association between each potential covariate with both the experience of induced abortion and past-year suicidal ideation as binary variables. Then using logistic regression analyses, we regressed each subject’s past-year suicidal ideation against induced abortion, after adjusting for covariates in a hierarchical manner. We included demographic covariates in model 1, with extra inclusion of psychosocial covariates in model 2, and added depression and anxiety disorders in model 3. We selected the covariates based on either literature or Chi-square test result with *p*-value < 0.1. We calculated the Nagelkerke / Cragg & Uhler’s pseudo R-squared as a comparison of model fit [46].

To further explore whether the association between induced abortion and past-year suicidal ideation differed among different groups, we performed subgroup analyses by age group, hometown, education attainment, length of stay in the city, attitude towards premarital pregnancy, the presence of clinically significant anxiety disorders and depression. Each time we added an interaction term to model 3 and reported the corresponding Odds ratio (OR) with 95% Confidence Interval (CI), as well as the *p*-value for overall interaction effect.

## Results

A total of 5578 unmarried female migrant workers agreed to participate in the study, with 5332 (95.6%) meeting the inclusion criteria and of them, 5115 (95.9%) completed the survey. Characteristics of the subjects were summarized in Table 1. The mean age was 23.3 ± 2.8 years (range = 18–35). Over 15% experienced induced abortion and over 8 % reported suicidal ideation during the past year.

**Table 1:** Characteristics of the study subjects (*N* = 5115)

Variables	n	%
City		
Shanghai	2255	44.1
Beijing	1638	32.0
Guangzhou	1222	23.9
Age (years)		
< 20	496	9.7
20–25	3353	65.6
> 25	1266	24.8
Highest education		
Middle school or below	1465	28.6
Senior high school	3233	63.2
College degree or above	417	8.2
Income (RMB-yuan**)**		
< 3200	1869	36.5
3200–4800	3099	60.6
> 4800	147	2.9
Hometown		
Urban	1253	24.5
Rural	3862	75.5
Years in the working place		
< 1	2237	43.7
1–5	611	12.0
> 5	2267	44.3
Tobacco use-past month		
No	4846	94.7
Yes	269	5.3
Alcohol use-past month		
No	4133	80.8
Yes	982	19.2
Daily Internet use-past month		
No	4555	89.1
Yes	560	10.9
Attitude toward premarital pregnancy		
Positive	1046	20.4
Negative	4069	79.6
Attitude toward multiple induced abortion		
Positive	156	3.0
Negative	4959	97.0
Rosenberg Self Esteem Scale		
High self-esteem (score > =15)	4514	88.3
Low self-esteem (score < 15)	601	11.7
GAD-anxiety		
Non-anxiety group (score < 10)	4506	88.1
Anxiety group (score > =10)	609	11.9
CESD-depression		
Non-depression group (score < 20)	3615	70.7
Depression group (score > =20)	1500	29.3
Have had premarital sex		
No	2142	41.9
Yes	2973	58.1
Have had unplanned pregnancy		
No	4296	84.0
Yes	819	16.0
Have had induced abortion		
No	4323	84.5
Yes	792	15.5
Have had suicidal ideation-past year		
No	4697	91.8
Yes	418	8.2
Have had suicidal plan-past year		
No	5032	98.4
Yes	83	1.6
Have had suicidal attempt-past year		
No	5064	99.0
Yes	51	1.0
Variables	Mean	Std.
Age	23.27	2.78
Rosenberg Self Esteem Scale	18.79	3.77
UCLS Loneliness Scale	4.46	3.94
GAD-anxiety	14.84	3.99
CESD-depression	16.75	9.35

The characteristics of the subjects with and without induced abortion, with and without past-year suicidal ideation were summarized in Table 2. Compared to those without induced abortion, those with the experience were more likely to be aged > 25, have lower education level but higher income, come from urban areas, have shorter stay in the working place, more likely to smoke, drink alcohol and surf the internet every day during the past month, have more positive attitude towards premarital pregnancy and multiple induced abortion, more likely to have low self-esteem and suicidal ideation during the past year. Compared to those without suicidal ideation, suicide ideators were more likely to have lower education and shorter stay in the working place, less likely to smoke but more likely to drink alcohol and report daily internet use, have more positive attitude towards premarital pregnancy and multiple induced abortion, more likely to have low self-esteem and experience loneliness, anxiety and depression, and have past experience of induced abortion.

**Table 2:** Characteristics of the subjects with/without induced abortion, and with/without past-year suicidal ideation (*N* = 5115)

Variables	With induced abortion (*n* = 792)	Without induced abortion (*n* = 4323)	*p*-value	With suicidal ideation (*n* = 418)	Without suicidal ideation (*n* = 4697)	*p*-value
Demographic characteristics						
Resident City						
Shanghai	344 (43.4%)	1911 (44.2%)	0.337	170 (40.7%)	2085 (44.4%)	0.292
Beijing	243 (30.7%)	1395 (32.3%)		138 (33.0%)	1500 (31.9%)	
Guangzhou	205 (25.9%)	1017 (23.5%)		110 (26.3%)	1112 (23.7%)	
Age (years%)						
< 20	52 (6.6%)	444 (10.3%)	0.004	49 (11.7%)	447 (9.5%)	0.220
20–25	530 (66.9%)	2823 (65.3%)		260 (62.2%)	3093 (65.9%)	
> 25	210 (26.5%)	1056 (24.4%)		109 (26.1%)	1157 (24.6%)	
Highest education						
Middle school or below	289 (36.5%)	1176 (27.2%)	< 0.001	155 (37.1%)	1310 (27.9%)	< 0.001
Senior high school	451 (56.9%)	2782 (64.4%)		230 (55.0%)	3003 (63.9%)	
College degree or above	52 (6.6%)	365 (8.4%)		33 (7.9%)	384 (8.2%)	
Income (RMB-yuan**)**						
< 3200	246 (31.1%)	1623 (37.5%)	< 0.001	168 (40.2%)	1701 (36.2%)	0.236
3200–4800	506 (63.9%)	2593 (60.0%)		237 (56.7%)	2862 (60.9%)	
> 4800	40 (5.1%)	107 (2.5%)		13 (3.1%)	134 (2.9%)	
Hometown						
Urban	218 (27.5%)	1035 (23.9%)	0.031	91 (21.8%)	1162 (24.7%)	0.176
Rural	574 (72.5%)	3288 (76.1%)		327 (78.2%)	3535 (75.3%)	
Years in the working place						
< 1	359 (45.3%)	1878 (43.4%)	0.101	217 (51.9%)	2020 (43.0%)	0.001
1–5	107 (13.5%)	504 (11.7%)		52 (12.4%)	559 (11.9%)	
> 5	326 (41.2%)	1941 (44.9%)		149 (35.6%)	2118 (45.1%)	
Psychosocial characteristics						
Tobacco use-past month						
No	726 (91.7%)	4120 (95.3%)	< 0.001	403 (96.4%)	4443 (94.6%)	0.110
Yes	66 (8.3%)	203 (4.7%)		15 (3.6%)	254 (5.4%)	
Alcohol use-past month						
No	557 (70.3%)	3576 (82.7%)	< 0.001	306 (73.2%)	3827 (81.5%)	< 0.001
Yes	235 (29.7%)	747 (17.3%)		112 (26.8%)	870 (18.5%)	
Daily Internet use-past month						
No	696 (87.9%)	3859 (89.3%)	0.250	345 (82.5%)	4210 (89.6%)	< 0.001
Yes	96 (12.1%)	464 (10.7%)		73 (17.5%)	487 (10.4%)	
Attitude toward premarital pregnancy						
Positive	321 (40.5%)	725 (16.8%)	< 0.001	108 (25.8%)	938 (20.0%)	0.004
Negative	471 (59.5%)	3598 (83.2%)		310 (74.2%)	3759 (80.0%)	
Attitude toward multiple induced abortion						
Positive	47 (5.9%)	109 (2.5%)	< 0.001	23 (5.5%)	133 (2.8%)	0.002
Negative	745 (94.1%)	4214 (97.5%)		395 (94.5%)	4564 (97.2%)	
Rosenberg Self Esteem Scale						
High self-esteem (score > =15)	679(85.7%)	3883 (89.8%)	0.017	310(74.2%)	4204(89.5%)	< 0.001
Low self-esteem (score < 15)	113(14.3%)	440 (10.2%)		108(25.8%)	493(10.5%)	
UCLS Loneliness Scale						
Low loneliness (score < =17%)	598 (75.5%)	3287 (76.0%)	0.748	196 (46.9%)	3689 (78.5%)	< 0.001
High loneliness (score > 17)	194 (24.5%)	1036 (24.0%)		222 (53.1%)	1008 (21.5%)	
Mental disorders						
GAD-anxiety						
Non-anxiety group (score < 10)	701 (88.5%)	3805 (88.0%)	0.694	333 (79.7%)	4173 (88.8%)	< 0.001
Anxiety group (score > =10)	91 (11.5%)	518 (12.0%)		85 (20.3%)	524 (11.2%)	
CESD-depression						
Non-depression group (score < 20)	494 (62.4%)	2757 (63.8%)	0.451	177 (42.3%)	3074 (65.4%)	< 0.001
Depression group (score > =20)	298 (37.6%)	1566 (36.2%)		241 (57.7%)	1623 (34.6%)	
Induced abortion						
No	–	–	–	310 (74.2%)	4013 (85.4%)	< 0.001
Yes	–	–		108 (25.8%)	684 (14.6%)	
Suicidal ideation-past year						
No	684 (86.4%)	4013 (92.8%)	< 0.001	–	–	–
Yes	108 (13.6%)	310 (7.2%)		–	–	

The association between induced abortion and suicidal ideation was summarized in Table 3. Induced abortion was associated with nearly twice the odds of having suicidal ideation across three models, with slight reduction in the effect size with extra adjustment of psychosocial covariates in model 2, and nearly no change with further adjustment of depression and anxiety in model 3. In model 3, higher education, longer stay in the working place and past-month smoking were associated with decreased, while past-month alcohol drinking and daily internet use, lower self-esteem, loneliness, depression and induced abortion with increased odds of suicidal ideation. The Nagelkerke R^2^ was 1.5% in the binary model for induced abortion, with the greatest increase (10.8%) with the introduction of psychosocial covariates.

**Table 3:** Multiple logistic regression of past-year suicidal ideation associated with induced abortion (*N* = 5115)

Variables	Suicidal ideation-past year OR (95% CI)		
	Model 1	Model 2	Model 3
Demographic characteristics			
*Age (years)*			
< 20	1	1	1
20–25	0.77(0.55–1.06)	0.77(0.55–1.08)	0.74(0.53–1.05)
> 25	0.93(0.65–1.34)	0.96(0.66–1.41)	0.96(0.65–1.41)
*Highest education*			
Middle school or below	1	1	1
Senior high school	0.66(0.54–0.83)**	0.70(0.56-0.88)**	0.70(0.56-0.88)**
College degree or above	0.73(0.49–1.08)	0.78 (0.51–1.19)	0.79(0.52–1.21)
*Years in the working place*			
< 1	1	1	1
1–5	0.82(0.60–1.13)	0.87(0.62–1.21)	0.85(0.61–1.19)
> 5	0.66(0.53–0.82)**	0.60(0.47-0.75)**	0.58(0.46-0.73)**
Psychosocial characteristics			
*Tobacco use-past month*			
No		1	1
Yes		0.22(0.12–0.39)**	0.21(0.12-0.38)**
*Alcohol use-past month*			
No		1	1
Yes		1.63(1.25–2.13)**	1.64(1.25-2.14)**
*Daily Internet use-past month*			
No		1	1
Yes		1.64 (1.21–2.22)**	1.63(1.20-2.21)**
*Attitude toward premarital pregnancy*			
Positive		1	1
Negative		0.89(0.69–1.16)	0.88(0.68–1.14)
*Attitude toward multiple induced abortion*			
Positive		1	1
Negative		0.71(0.43–1.18)	0.75(0.45–1.24)
*Rosenberg Self Esteem Scale*			
High self-esteem (score > =15)		1	1
Low self-esteem (score < 15)		1.95(1.49–2.55)**	1.85(1.39-2.45)**
*UCLS Loneliness Scale*			
Low loneliness (score < =17)		1	1
High loneliness (score > 17)		3.64(2.92–4.53)**	3.17(2.49-4.04)**
Mental disorders			
*GAD-anxiety*			
Non-anxiety (score < 10)			1
Anxiety (score > =10)			0.85(0.62–1.16)
*CESD-depression*			
Non-depression (score < 20)			1
Depression (score > =20)			1.50(1.16–1.93)**
*Induced abortion*			
No	1	1	1
Yes	1.97(1.56–2.50)**	1.89(1.46-2.45)**	1.89(1.46-2.44)**
*Nagelkerke R Square*	0.028	0.136	0.140

**P < 0.05

The results for our subgroup analyses were summarized in Table 4. The association between induced abortion and suicidal ideation were significant among those aged > 20 with larger effect among those aged > 25, those with a length of stay of < 1 year or > 5 years with larger effect in the latter, and in the non-anxiety group and non-depression group. We did not find different effect associations by hometown, education level or attitude towards premarital pregnancy.

**Table 4:** Subgroup analyses of past-year suicidal ideation associated with induced abortion (*N* = 5115)

Variables	n	OR^a^	95% CI	*p*-value for interaction
*Age (years)*				
< 20	496 (9.7%)	0.75	0.27–2.11	0.005
20–25	3353 (65.6%)	1.58	1.14–2.19	
> 25	1266 (24.8%)	3.37	2.16–5.28	
*Hometown*				
Urban	1253 (24.5%)	1.52	0.90–2.57	0.351
Rural	3862 (75.5%)	2.01	1.51–2.69	
*Highest education*				
Middle school or below	1465 (28.6%)	1.75	1.17–2.62	0.891
Senior high school	3233 (63.2%)	1.98	1.40–2.79	
College degree or above	417 (8.2%)	2.03	0.80–5.18	
*Years in the working place*				
< 1	2237 (43.7%)	1.54	1.06–2.25	0.008
1–5	611 (12.0%)	0.96	0.46–1.99	
> 5	2267 (44.3%)	2.98	2.02–4.39	
*Attitude toward premarital pregnancy*				
Positive	1046 (20.4%)	1.50	0.97–2.31	0.195
Negative	4069 (79.6%)	2.14	1.56–2.92	
*GAD-anxiety*				
Non-anxiety (score < 10)	4506 (88.1%)	2.28	1.74–3.00	0.001
Anxiety (score > =10)	609 (11.9%)	0.64	0.31–1.30	
*CESD-depression*				
Non-depression (score < 20)	3615 (70.7%)	2.94	2.08–4.15	< 0.001
Depression (score > =20)	1500 (29.3%)	1.20	0.83–1.74	

^a^Adjusting for all the other covariates in model 3

## Discussion

To our knowledge, this was the first study targeting specifically at the association between induced abortion and suicidal ideation among unmarried female migrant populations working in urban cities in China, who are an important workforce in the national urbanization process but have received insufficient attention with regards to their mental health. We found nearly twice the odds of past-year suicidal ideation associated with the experience of induced abortion, which was independent of demographic, psychosocial and mental disorder characteristics. The association was stronger among those aged over 25, having stayed in the working place for over 5 years, and without depression or anxiety disorders.

The prevalence of past-year suicidal ideation in our subjects, 8.2%, was higher than 6.5% among rural female populations aged 16–34 [33], the lifetime suicidal ideation of 3.9% among the general population [47] and 5.8% among the urban dwellers of similar age range in China [10]. It is much higher than the 2.8% lifetime suicidal ideation prevalence reported among male migrant workers in railway construction site in China [48]. Higher rate of suicide and suicide attempt have been reported among migrants in Europe despite large heterogeneity for different migrant groups [1, 49]. Nearly 30 % of our study subjects experienced depression, higher than that reported in other studies of Chinese migrant workers [17, 50, 51], and the prevalence of anxiety was similar to previous report [50].

The positive association between induced abortion and suicidal ideation was consistent with previous studies. The first cohort study using national registry data in Finland reported over 3 times increased risk of suicide following induced abortion compared with the general population, which corresponded to nearly 6 times the risk compared to birth givers [52], and the higher risk even persists until now since the initiation of the Current Care Guideline in Finland which highlights monitoring the mental disorders of women during the post-termination window [53]. Positive associations between suicidal ideation and induced abortion were reported in longitudinal studies [54, 55]. In China, only one study reported no effect of abortion on suicidal ideation but negative effect on suicide acceptability among females aged 15–34 in rural areas [56]. Our study adds to the evidence of induced abortion-suicidality association and highlights the mental health issues among unmarried female migrant workers.

In our hierarchical analyses, induced abortion was associated with increased odds for suicidal ideation in all of the models. It was reported that migrant population in China have slight advantage in mental well-being than the general population [9, 17] but they are characterized by lower level of medical service utilization especially mental health services [51]. With little knowledge of contraception, and further complicated by the one-child policy implemented since 1978, as well as the differentiated maternal health care practiced in urban cities toward local dwellers and migrant females, unplanned pregnancies predominantly end up with induced abortion among unmarried migrant female workers. In our data, the majority (96.9%) of those having unplanned pregnancy underwent induced abortion. Induced abortion might be a natural choice for them but the consequence of abortion might have more lasting and complex impact on their emotion and mental well-being. The current maternal care system in urban cities can only trace reported pregnancy with a complete records of medical examination during pregnancy, which is less capable of capturing induced abortion and managing the post-termination health issues of the unmarried female population experienced with induced abortion.

In the final model, induced abortion and other behavioral and psychosocial characteristics overtook depression with relation to suicidal ideation, and anxiety was no longer a significant predictor. Also contrary to western studies [54, 55, 57], we did not find associations between induced abortion and either depression or anxiety, which suggested some distinct links between induced abortion and suicidality among our population. Based on some overlapping characteristics of the subjects with induced abortion and suicidal ideation shown in our binary analyses(i.e., lower education, shorter stay in the working place, more alcohol consumption and internet use, more tolerant attitude towards primatial sex and multiple abortion, more likely to have low self-esteem), we could portrait a group of subjects who were less behaviorally strict but not necessarily more depressed or disturbed by mental disorders, and had lower level of social support which might in turn drive them to seek sensational or other problem behaviors. The link between the suicidal ideation and induced abortion among this population may not be mediated by depression, but more likely, an indication of some personality and temperament. Social support may play an important role here since the decision to continue or to give up the birth might be directly related to the perceived social support [58], and interpersonal connectedness is one of the core aspects of suicidality [59, 60]. Among common theories in the explanation between abortion and mental distress [55], stress coping may play an essential role here due to the loose link between abortion and depression and anxiety, but the high prevalence of risky behaviors which may signify mal-coping strategies.

Despite that only 5.3% of the subjects reported tobacco use during the past month, the significant negative association between smoking and suicidal ideation warrant further research. Potential underreport of tobacco use could bias the association, or maybe smoking was regarded as a means for relieving the pressure among our subjects, which reduced their risk for suicidal ideation. The large increase in the Nagelkerke R^2^ (10.8%) in model 2 highlights the psychosocial determinants of suicidality. In general, risk prediction for suicide is unsatisfactory [61], and the R^2^ of 14.0% in our final model was acceptable.

There seem to be stronger association between induced abortion and suicidal ideation with older age. It may be due to stronger emotional loss caused by fetal loss as age grows, or a manifestation of the accumulation of psychosocial stress overtime. Meanwhile, although longer stay in the working place was protective for suicidal ideation, the stronger association between abortion and suicidal ideation among those with longer stay is suggestive of the strengthening of some personality or propensity overtime. The stronger association among those without anxiety or depression further corroborates our inference that induced abortion was associated with suicidality independent of mental disorders among this population. Interestingly, among the anxiety group, induced abortion was associated with reduced odds of suicidal ideation despite the lack of power in revealing significance. A possible explanation might be that the termination was a relief to those unprepared mother-to-bes which reduced their psychological burdens.

Our results should be interpreted considering the following limitations. Above all, we cannot draw causal conclusions due to the cross-sectional design. However, suicidal ideation measured within the past year and induced abortion measured within lifetime partly reduced reversal causality. Second, the clustered convenient sampling method brings selection bias although it is the most common method used for recruiting migrant workers who are usually highly aggregated. Third, we did not discriminate between single-time abortion and multiple induced abortions, which may have different associations with suicidal ideation. Fourth, we did not measure potential confounders such as interpersonal violence, childhood experience, working environment, physical diseases and the experience of health seeking behavior, and we may have underestimated depression prevalence without asking antidepressant use. Fifth, we compared suicidal ideation between women with induced abortion and those without such experience most of whom we assumed to be never pregnant, but not ideally two groups of pregnant women with one experiencing induced abortion and the other delivering the baby. Nevertheless, the large sample size, the investigation in the three biggest cities in China with a wide geographic coverage, the structured questionnaire used across the three sites and the inclusion of a wide spectrum of covariates in the survey constituted the major strengths of our study.

## Conclusions

Lifetime induced abortion was associated with nearly twice the odds for suicidal ideation during the past year among unmarried female migrant workers in metropolitan cities in China. An improvement of mental health of the population requires policy change, medical system support, enhanced communication between the service seekers and health care providers, and importantly, the establishment of an enabling environment to strengthen social support and facilitate help seeking behaviors of those less advantaged population.

## Abbreviations

CDC: Center for Disease Control and Prevention
CESD: Center for Epidemiologic Studies Depression Scale
CI: Confidence interval
GAD: Generalized Anxiety Disorder
OR: Odds ratio
WHO: World Health Organization
